# Does Tooth Position Change Between Preparation and Cementation in Overlay Restoration? A Clinical Evaluation With/Out Interappointment Provisionalization

**DOI:** 10.1111/jerd.70051

**Published:** 2025-10-24

**Authors:** Del Bianco Federico, Mancuso Edoardo, Mazzitelli Claudia, Maravic Tatjana, Mazzoni Annalisa, Breschi Lorenzo

**Affiliations:** ^1^ Department of Biomedical and Neuromotor Science (DIBINEM) Alma Mater Studiorum, University of Bologna Bologna Italy

**Keywords:** digital scan, indirect partial restoration, overlay, temporary restoration, tooth position

## Abstract

**Objective:**

To evaluate whether teeth prepared for overlay restoration, as well as their antagonist or adjacent teeth, undergo positional changes between tooth preparation and final cementation appointments, with or without the use of a temporary restoration.

**Materials and Methods:**

Fifty teeth (maxillary and mandibular molars and premolars) requiring overlay restorations were prepared and randomly assigned to 2 groups, according to whether a temporary restoration (Telio, Ivoclar Vivadent) was placed during the interval between appointments or not. Digital scans (Trios3) were obtained at the first appointment, immediately after tooth preparation (T0), and at the second appointment, before cementation (T1). The T0 and T1 .STL files were superimposed (Geomagic), and linear displacements of the abutment tooth (AB), its antagonist (AN), and the adjacent tooth (AD) were measured, providing a quantitative assessment of positional changes. Maximum tooth movement distances were statistically analyzed (*p* < 0.05).

**Results:**

The factor “tooth position” significantly influenced maximum tooth movement distance (*p* < 0.0001). Specifically, AB demonstrated greater positional changes compared to AN (*p* < 0.0001) and AD (*p* < 0.0001). Moreover, the placement of a temporary restoration significantly reduced the extent of tooth movement in maxillary molars (*p* = 0.002) but not in other investigated tooth classes (*p* > 0.05).

**Conclusions:**

Omission of a temporary restoration during the interval between tooth preparation and overlay cementation may increase the risk of tooth positional changes for specific tooth classes.

## 1 Introduction

Indirect overlay restorations are widely used in contemporary restorative dentistry for the conservative rehabilitation of structurally compromised posterior teeth [1]. Their success depends on precise tooth preparation, accurate impression taking, and correct material selection [2, 3, 4]. This clinical workflow typically involves a two‐visit protocol: during the first appointment, the tooth is prepared and an impression (digital or conventional) is taken and sent to the dental technician; during the second appointment, the definitive restoration is delivered, tried in, and cemented. In the period between these two visits—commonly ranging from several days to a few weeks—the prepared tooth may lose not only its occlusal contacts with the antagonist dental element but also proximal contacts with adjacent teeth [5]. The stable maintenance of the spatial relationships within the dental arch during the interval between preparation and final cementation is imperative for the correct fitting of the restoration before delivery [6, 7].

Tooth position in the dental arch is maintained through a dynamic balance of forces: occlusal contacts stabilize vertical dimension, while proximal contacts preserve mesio‐distal positioning [8, 9, 10]. The loss of these stabilizing contacts, as observed in cases of extractions, trauma, or extensive decay, has been previously documented to cause pathologic migration, tipping, rotation, or supraeruption of teeth [11, 12, 13]. Periodontal status influences the magnitude of tooth movement, whereas mechanical stresses simultaneously initiate cellular responses that modulate bone resorption and thereby affect tooth displacement [12, 13, 14]. Orthodontic literature has also demonstrated that teeth tend to move when left without contacts or even under low‐magnitude, sustained forces [15]. However, to date, it is still unclear whether posterior teeth prepared for overlay restorations exhibit similar tendencies for positional change during the provisional phase.

From a clinical perspective, the risk of unwanted tooth movement—or, in some cases, esthetic concerns—has traditionally led many practitioners to adopt provisional restorations placement after tooth preparation, maintaining them throughout the interappointment period until delivery of the definitive overlay [16, 17]. The use of such temporaries for indirect partial restorations has rarely been questioned, although some clinicians regard them as time‐consuming, particularly during removal prior to cementation, and not cost‐effective. Consequently, a portion of practitioners prefer to avoid placing an interappointment provisional [18]. This trend is especially evident in the current era of digital dentistry, where restorations can often be fabricated and delivered within a short timeframe [19]. Skipping provisionalization can simplify clinical procedures, reduce the risk of contamination at the adhesive interface, and save chairside time [20]. However, leaving the prepared tooth without a provisional restoration exposes it to structural and sensitivity issues [18, 21], as well as makes it vulnerable to unmonitored spatial changes [21]. Even minor positional shifts—such as mesial drift, extrusion, or rotation—may compromise the fit of the definitive restoration, potentially requiring occlusal adjustments or, in some cases, remanufacture of the prosthesis [22, 23, 24, 25]. Such outcomes negatively affect treatment duration, cost‐efficiency, and overall patient satisfaction [18].

To date, standardized protocols for evaluating dental movement in the time interval between tooth preparation and final cementation are lacking [26]. Most clinical studies have relied on indirect methods, which are often limited by low precision, lack of qualitative detail, and subjectivity. Intraoral scanners (IOSs) have recently been introduced as a promising alternative to overcome these limitations [27]. IOS technology has demonstrated high sensitivity in detecting tooth movements [18] and has shown potential in offering a more systematic and reproducible approach for evaluating dental displacement in clinical settings [28].

As overlay restorations are increasingly favored for their minimally invasive design and ability to preserve sound tooth structure, establishing a reliable and evidence‐based protocol for managing the interappointment phase is of growing importance. Identifying the presence or absence of tooth migration may provide crucial information to guide clinical decision‐making in restorative dentistry.

The aim of this study was to evaluate whether measurable changes in tooth position occur in maxillary and mandibular molars and premolars prepared for overlay restorations during the time between preparation and final cementation, as well as in adjacent and antagonist teeth. The analysis also aimed to assess whether the interappointment provisionalization influences such movements. Specifically, the research hypotheses were that (1) teeth prepared for overlay restorations, as well as their adjacent and antagonist teeth, change position between clinical appointments, and that (2) the presence of a temporary restoration influences teeth movements between clinical appointments.

## 2 Materials and Methods

### 2.1 Ethical Approval and Administrative Procedures

This was an observational, single‐site study that was conducted at the Department of Restorative Dentistry of the Dental Clinic of Bologna University (DIBINEM), Bologna, Italy, in the period from March 2025 until July 2025. Before initiating the trial, the research protocol was reviewed and approved by the University Ethics Committee (No.: 730‐2024‐OSS‐AUSLBO) and the study was conducted in accordance with the Helsinki Declaration of Human Rights [29]. Patients attending the Dental Clinic who met the inclusion criteria (as detailed below) were informed about the purpose, methodology, and implications of the study. An information sheet outlining all relevant aspects of the research was provided and explained in detail. Upon agreeing to participate, each patient was required to sign a written informed consent form. Participation in the study was entirely voluntary and did not involve any form of financial compensation.

### 2.2 Sample Size Determination, Patient Recruitment and Eligibility Criteria

The number of patients to be enrolled in the study was determined by means of a statistical power analysis using G*Power (Version 3.1.5, Germany) based on the effect size (*f* = 0.6158088) reported in a previous study [30]. Assuming a significance level (*α*) of 0.05 and a statistical power (1 − *β*) of 0.90, the minimum required sample size was calculated to be 10 participants per group.

Male and female subjects between 18 and 80 years of age, in good general and oral health, requiring an overlay restoration on a posterior maxillary or mandibular tooth, and presenting at least one healthy antagonist tooth and one adjacent tooth (mesial and/or distal) were considered eligible for inclusion. Additional inclusion criteria required the absence of temporomandibular joint disorders, parafunctional habits, or other dental limitations that could affect the outcome of the treatment.

Exclusion criteria included patients who refused to sign the informed consent form or declined to undergo the proposed restorative treatment, teeth with mobility greater than Grade 2, teeth with structural loss exceeding 75%, and patients currently undergoing orthodontic treatment.

A total of 42 participants who met the inclusion criteria were finally enrolled in the study. Participant characteristics—including age, sex, type of tooth (molar or premolar), and the location of the restored tooth within the dental arch (maxillary or mandibular)—were collected. After overlay cavity preparation, participants were randomly allocated into two experimental groups (*n* = 25 teeth prepared for an overlay restoration per group) using a 1:1 computer‐generated randomization protocol (Microsoft Excel). In Group 1, no provisional restoration was placed between the preparation of the abutment and the final overlay cementation. In Group 2, a provisional restoration (Telio, Ivoclar Vivadent, Schaan, Liechtenstein) was placed to maintain both occlusal and interproximal contacts. Participants who lost their provisional restorations prior to the cementation appointment were excluded from the analysis.

**FIGURE 1:**
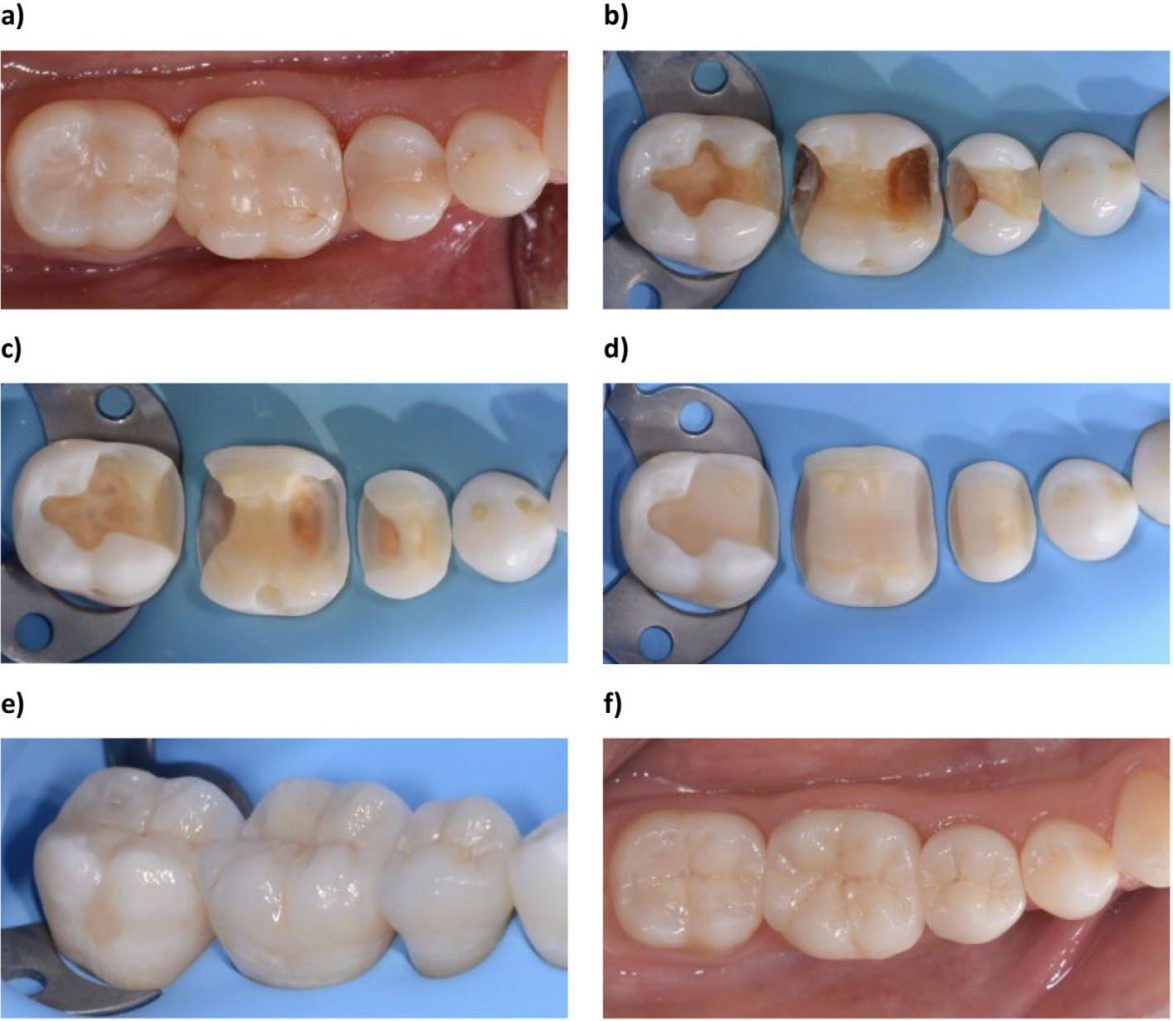
Treatment of lower right quadrant with indirect lithium disilicate partial restorations: (A) Initial situation with incongruous composite fillings on 4.5, 4.6, and 4.7 and underneath secondary caries; (B) after removal of the old restoration; (C) after cavity cleaning; (D) resin composite build‐ups and final preparations; (E) cementation of lithium disilicate restorations; and (F) 1 year follow‐up.

Tooth preparations were carried out following the clinical guidelines recommended for the fabrication of lithium disilicate restorations: 1.5 mm occlusal reduction, 1 mm axial wall reduction, rounded internal angles, and a 6° taper (Figure 1A–F) [31]. All preparations were performed by a single, trained operator with more than 10 years of clinical experience (F.D.B.) using ×4.5 magnification to verify reduction parameters with a silicone index (Elite HD+; Zhermack), a CP‐15 periodontal probe, and a red‐ring contra‐angle handpiece equipped with a diamond rotary instrument (Neodiamond; Microcopy Dental).

**FIGURE 2:**
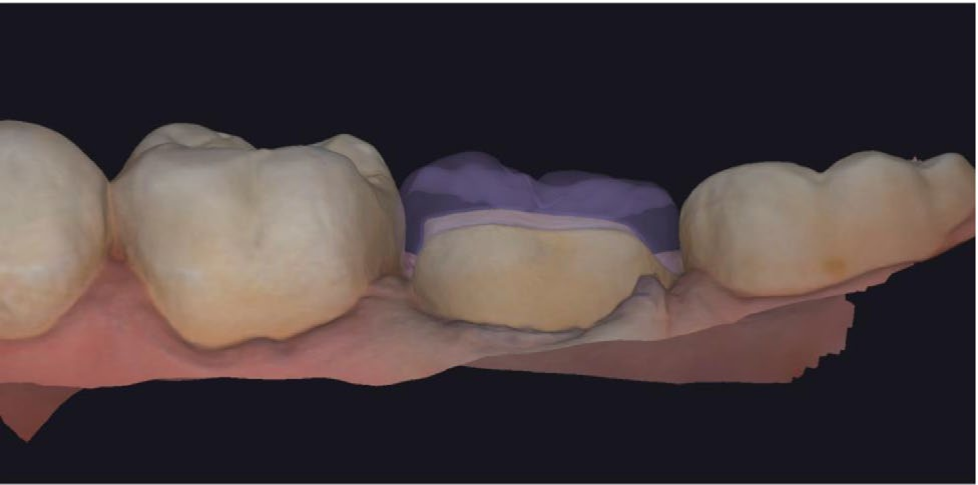
Tooth preparation and overlay design based on the scan acquired at the first appointment after cavity preparation for the indirect partial restoration. Even slight deviations from this position may cause difficulties in seating the final restoration, which in some cases can be resolved with minor chairside adjustments, but in more critical situations may require replacement of the entire restoration. In both scenarios, additional time and costs are involved.

Following preparation, digital impressions of both arches and bite registrations were acquired using an IOS with a precision of 4.5 ± 0.9 μm (TRIOS 3; 3Shape, Copenhagen, Denmark), and the datasets were exported as .STL files (T0). A second digital impression (T1) was obtained on the day of cementation, following removal of the provisional restoration in Group 2 (Figure 2) [3, 32, 33].

### 2.3 Software Analysis

For each sample, the .STL files corresponding to timepoints T0 and T1 were superimposed using Geomagic Qualify 12 (Geomagic, North Carolina, USA) [7]. Initial alignment was performed using the software's “best‐fit alignment” function. The gingival portion of the 3D models was subsequently removed to isolate the clinical crowns. A second, refined alignment was then carried out using the “N‐point alignment” method, which involved selecting stable reference points on teeth distant from the abutment to reduce alignment bias.

**FIGURE 3:**
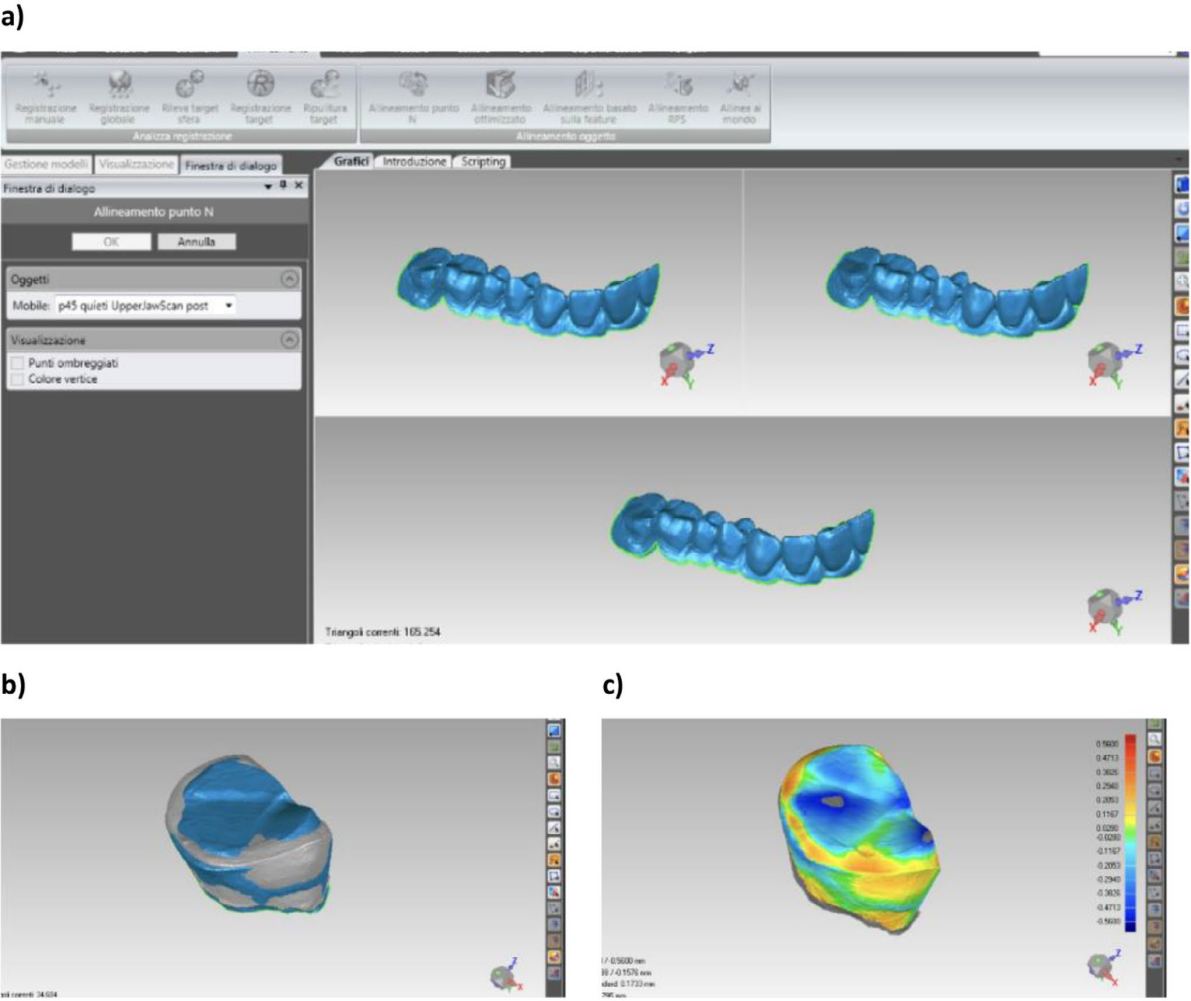
Measurement of position changes using Geomagic software: (A) N‐point alignment of two intraoral scans; (B) matching of the two scans per each tooth; and (C) measurement of maximum distance between the two scans with warmer colors indicating higher discrepancies.

After alignment, four specific teeth were segmented from both T0 and T1 models: the abutment (AB), its antagonist (AN), and the mesial and distal adjacent tooth (AD). For each of these, the software computed the mean distance between the T0 and T1 surfaces. These distances reflect linear displacements calculated between corresponding surface points across the two timepoints (T0 to T1), providing a quantitative measure of positional changes (Figure 3A,B) [34].

### 2.4 Statistical Analysis

Data on maximum distance of the tooth movement was statistically analyzed using a linear mixed model, after the verification of normal distribution and equal variance (Shapiro–Wilk and Brown–Forsythe test; *p* > 0.05). The analysis investigated the effects of several factors: “provisionalization” (yes/no), “tooth position” (abutment/adjacent/antagonist), and “tooth class” (maxillary molars—MXM, mandibular molars—MNM, maxillary premolars—MXP, mandibular premolars—MNP, maxillary canines—MXC, mandibular canines—MNC) on the maximum distance of the tooth movement. Bonferroni correction was used for post hoc analyses. The significance threshold was set at *p* < 0.05, and all the analyses were performed in IBM SPSS v. 25 (IBM, Armonk, NY, USA).

## 3 Results

A total of 50 restorations were performed on 42 patients. Mean age, sex distribution of the participants, and mean number of days between the appointments are presented in Table 1. The distribution of different tooth types, including abutment, adjacent, and antagonist teeth within the groups of patients that did or did not receive a provisional crown is shown in Table 2. The overlays were placed primarily on molars (80%).

**TABLE 1:** Details of the patients involved in the study and the number of days elapsed between the two appointments.

	Patients (*n*)	Overlays (*n*)	Age (mean ± SD)	Sex (M/F)	Days passed between appointments (mean ± SD)
Provisional crown, yes	20	25	55.8 ± 11.5	4/16	7.9 ± 2.3
Provisional crown, no	22	25	55.1 ± 11.7	15/7	8.8 ± 5.3

**TABLE 2:** Number and position of the classes of teeth included in the study.

	Abutment	Adjacent	Antagonist	Total
Provisional crown, yes				
Maxillary molars	8	5	10	23
Mandibular molars	13	16	7	36
Maxillary premolars	1	7	4	12
Mandibular premolars	3	8	1	12
Maxillary canines	0	1	0	1
Mandibular canines	0	1	0	1
Provisional crown, no				
Maxillary molars	8	10	10	28
Mandibular molars	11	10	8	29
Maxillary premolars	3	8	3	14
Mandibular premolars	3	5	3	11
Maxillary canines	0	1	0	1
Mandibular canines	0	1	0	1
Total	50	73	46	169

The linear mixed model analysis demonstrated that “tooth position” influenced maximum tooth movement distance (*p* < 0.0001), while “provisionalization” (*p* = 0.517) or the interaction of the two factors did not influence this distance (*p* = 0.805). Furthermore, “tooth class” (*p* = 0.002), as well as the interaction between the “tooth class” and “provisionalization” (*p* = 0.014) significantly influenced the extent of tooth movement (Table 3). The post hoc analysis demonstrated that the abutment tooth had the tendency to change position more than the antagonists (*p* < 0.0001) or the adjacent teeth (*p* < 0.0001; Table 4). Further, maxillary molars changed their position significantly less if a provisional crown was placed on the abutment tooth (*p* = 0.002). Also, maxillary molars demonstrated a larger maximum tooth movement distance compared to maxillary (*p* = 0.001) and mandibular premolars (*p* = 0.005) when a provisional crown was not placed on the abutment tooth (Table 5).

**TABLE 3:** Fixed effects table for the linear mixed model analysis (dependent variable tooth movement—mm).

Source	Num. df	Den. df	*F*	*p*
Intercept	1	170.004	147.605	< 0.0001
Provisionalization	1	170.004	0.422	0.517
Tooth position	2	170.004	13.135	< 0.0001
Tooth class	5	170.004	3.996	0.002
Provisionalization × tooth position	2	170.004	0.217	0.805
Provisionalization × tooth class	5	170.004	2.933	0.014
Sex	1	170.004	0.034	0.853

**TABLE 4:** Table expressing maximum tooth movement distance (mean ± standard error) in the different investigated groups divided by tooth position and provisionalization.

	Provisional crown, yes	Provisional crown, no
Abutment	0.41 ± 0.03^Aa^	0.41 ± 0.03^Aa^
Adjacent	0.29 ± 0.02^Ab^	0.3 ± 0.03^Ab^
Antagonist	0.25 ± 0.03^Ab^	0.29 ± 0.03^Ab^

*Note*: Different superscript uppercase letters demonstrate statistically significant differences within rows, while lowercase letters demonstrate differences within columns (*p* < 0.05).

**TABLE 5:** Table expressing maximum tooth movement distance (mean ± standard error) in the different investigated groups divided by tooth class and provisionalization.

	Provisional crown, yes	Provisional crown, no
Maxillary molars	0.29 ± 0.03^Ba^	0.42 ± 0.03^Aa^
Mandibular molars	0.38 ± 0.02^Aa^	0.35 ± 0.03^Aab^
Maxillary premolars	0.23 ± 0.04^Aa^	0.21 ± 0.04^Ab^
Mandibular premolars	0.27 ± 0.04^Aa^	0.24 ± 0.04^Ab^
Maxillary canines	0.39 ± 0.14^Aa^	0.17 ± 0.1^Aab^
Mandibular canines	0.19 ± 0.14^Aa^	0.3 ± 0.11^Aab^

*Note*: Different superscript uppercase letters demonstrate statistically significant differences within rows, while lowercase letters demonstrate differences within columns (*p* < 0.05).

## 4 Discussion

The aim of this study was to evaluate whether tooth movements occur during the preparation and delivery phases of lithium disilicate overlays, specifically involving abutment, antagonist, and adjacent teeth. According to the results, the first research hypothesis must be accepted. While adjacent and antagonist teeth did not exhibit substantial displacements, abutment teeth showed significant positional changes between the preparation stage and the delivery of the definitive restoration (Table 4). Moreover, it was demonstrated that certain tooth classes were more prone to positional changes than others, suggesting a non‐uniform pattern of movement (Table 5).

Tooth movement is a biologically driven process that occurs when mechanical forces are applied to the dentition, leading to adaptive changes in the supporting tissues [9]. Teeth, unless ankylosed, are never completely immobile within their sockets but rather maintain a dynamic equilibrium influenced by occlusal, interproximal, and periodontal forces [12]. This explains why the loss of contact with adjacent or antagonistic teeth—such as after extraction or orthodontic movement—often results in spontaneous drifting, extrusion, or tipping, as teeth naturally seek to re‐establish stable occlusal and proximal relationships [8]. Periodontal conditions play a central role in this process: [12] molars with reduced periodontal support demonstrate significantly greater vertical displacement compared with periodontally healthy teeth (1.97 vs. 0.48 mm, respectively) [14]. Similarly, the absence of antagonistic or adjacent contacts has been shown to promote clinically relevant movements, both vertical and rotational, with progressive closure of extraction spaces over time [12, 14]. At the cellular level, mechanical stress alters vascular supply and induces the release of neurotransmitters, cytokines, and growth factors within the periodontal ligament, creating a microenvironment conducive to bone resorption or apposition [9]. The metabolic changes secondary to the rapid periodontal response are mainly responsible for tooth movements. Recently, it has been observed that the rubber dam tension maintained during intraoral scan could interfere with the stability of the prepared tooth (in the case of the study, first molars were taken into consideration), thus influencing the interproximal fitting of the indirect partial restorations [35].

**FIGURE 4:**
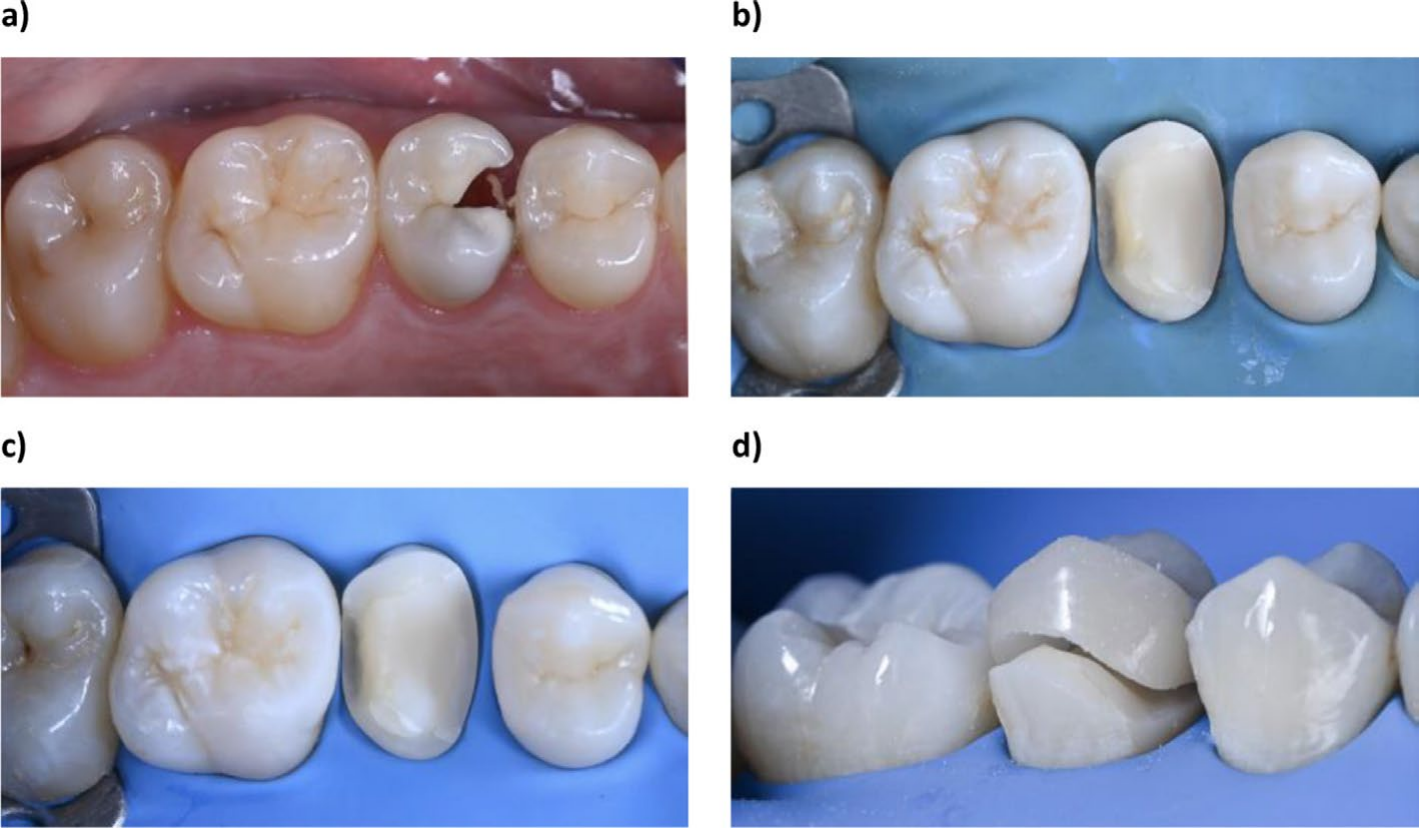
Case of interappointment tooth movement when no temporary filling was used: (A) Initial situation showing the presence of caries lesion with the loss of the mesial interproximal wall of the upper right second premolar; (B) during the first appointment, the caries lesion was removed, the resin composite build‐up was created and the tooth was prepared and scanned; (C) after 2 weeks, the rubber dam was placed and it was possible to observe that the tooth moved significantly in a distal direction; and (D) as a consequence, during the try‐in procedure, the restoration could not properly be seated.

These well‐documented biological mechanisms provide a rationale for the positional changes observed in abutment teeth during the relatively short interval between cavity preparation and restoration delivery, as observed in this study (Table 3). Even minor vertical or rotational shifts can compromise the accuracy of fit, potentially leading to seating difficulties at the delivery appointment (Figure 4A–D). This clinical challenge has been increasingly acknowledged, as improper fitting of partial indirect restorations not only complicates cementation but has also been associated with an increased risk of secondary caries in adjacent teeth due to altered contact morphology [6]. From a clinical point of view, this problem could be overcome with the placement of a temporary filling material that will serve to “fill” and “lock” the spaces.

In this regard, the second research hypothesis must therefore also be supported, as the placement of an interappointment provisional restoration was shown to influence tooth movement, with the magnitude of this effect varying by tooth class. Specifically, molars were more prone to positional changes compared to both maxillary and MNP, and MXP were the most affected by lack of provisionalization (Table 5).

Provisional materials are typically designed to restore proximal and occlusal contacts, thereby maintaining positional stability of the prepared tooth [20]. While this approach could be effective in limiting undesirable movement, it also carries well‐recognized drawbacks: increased chairside time, added material costs, and difficulties in removal that may negatively impact adhesive procedures by reducing wettability and bond strength [22, 23, 24, 25]. Over a plethora of materials available on the market, recently, resin‐based, eugenol‐free provisional materials are generally preferred, as they combine satisfactory esthetic and functional performance with compatibility for adhesive luting [18].

Nevertheless, clinical strategies have evolved to minimize or bypass the need for provisionalization. One notable example is the immediate dentin sealing (IDS) technique, in which an adhesive system is applied immediately after tooth preparation and before impression taking [22]. IDS has been associated with improved bacterial sealing, reduced postoperative sensitivity, and enhanced hybridization of dentin, with benefits reported for both vital and endodontically treated teeth [21, 36]. While promising, IDS does not address the risk of spatial changes in abutment position, which remains a potential source of misfit in indirect adhesive restorations.

From a clinical standpoint, the findings suggest that provisionalization should not be dismissed solely as a matter of convenience. Difficulties encountered during the seating of definitive restorations raise legitimate concerns about unmonitored positional shifts of unprotected abutments between appointments. In this respect, interappointment provisional restorations may serve not only as a protective and esthetic solution but also as a preventive measure to preserve spatial stability, thereby supporting the predictability and longevity of indirect adhesive restorative treatments.

This study provides just preliminary evidence that both biological and procedural factors may contribute to tooth movement during the restorative workflow of lithium disilicate overlays. The results must, however, be interpreted with caution, as some limitations can be addressed. The limited number of restorations analyzed and the distribution of tooth classes may have influenced statistical power, with an abundance of molars compared to other tooth classes. Further, the exclusion of participants who lost their temporary restorations before the final appointment could have created selection bias. It is probable that patients who lose their temporary restorations are more likely to engage in certain habits, such as parafunctional activity or a particular diet, which may contribute to tooth movement. By eliminating these patients, the study could have underestimated the true level of positional changes in the provisionalization group in a real‐world clinical environment. However, this was necessary to standardize as much as possible the conditions under which tooth movements were investigated and avoid important variability and discrepancies in the results. Finally, the method used for tooth movement assessment does not allow for a critical qualitative analysis. The clinical relevance of tooth movement is frequently associated with its direction (e.g., vertical supraeruption, mesial/distal drift, or rotation), rather than its total amount, which was not possible to determine.

Future research efforts should be made toward clinical studies of a larger scale, with operators of different clinical experience, and toward the development of the assessment method that would provide both qualitative and quantitative data.

## 5 Conclusions

Within the limitations of this current study, it was concluded that

1. Teeth prepared for overlay restorations changed their position more significantly compared to their adjacent or antagonist teeth.
2. The presence of a temporary restoration influenced tooth movements between the clinical appointments only in MXM.

## Author Contributions

**Del Bianco Federico**, **Mancuso Edoardo**, and **Mazzitelli Claudia:** conceptualization. **Del Bianco Federico**, **Mancuso Edoardo**, **Mazzitelli Claudia**, and **Maravic Tatjana:** methodology. **Mancuso Edoardo:** data curation. **Del Bianco Federico**, **Mancuso Edoardo**, **Mazzitelli Claudia**, and **Maravic Tatjana:** formal analysis and investigation. **Mancuso Edoardo** and **Maravic Tatjana:** software. **Mazzitelli Claudia** and **Maravic Tatjana:** validation. **Mazzitelli Claudia**, **Mazzoni Annalisa**, and **Breschi Lorenzo:** project administration. **Mazzoni Annalisa** and **Breschi Lorenzo:** visualization. **Mancuso Edoardo**, **Mazzitelli Claudia**, and **Maravic Tatjana:** writing – original draft preparation. **Mazzitelli Claudia**, **Maravic Tatjana**, **Breschi Lorenzo**, and **Mazzoni Annalisa:** writing – review and editing.

## Disclosure

The authors have nothing to report.

## Conflicts of Interest

The authors declare no conflicts of interest.

## Data Availability

The data that support the findings of this study are available from the corresponding author upon reasonable request.
